# Effect of Impurities on the Decarbonization of Calcium Carbonate Using Aqueous Sodium Hydroxide

**DOI:** 10.1021/acssuschemeng.2c02913

**Published:** 2022-08-26

**Authors:** Marco Simoni, Theodore Hanein, Chun Long Woo, John Provis, Hajime Kinoshita

**Affiliations:** Department of Materials Science & Engineering, University of Sheffield, S1 3JD Sheffield, United Kingdom

**Keywords:** decarbonization, CO_2_ sequestration, ambient conditions, cement, CaCO_3_

## Abstract

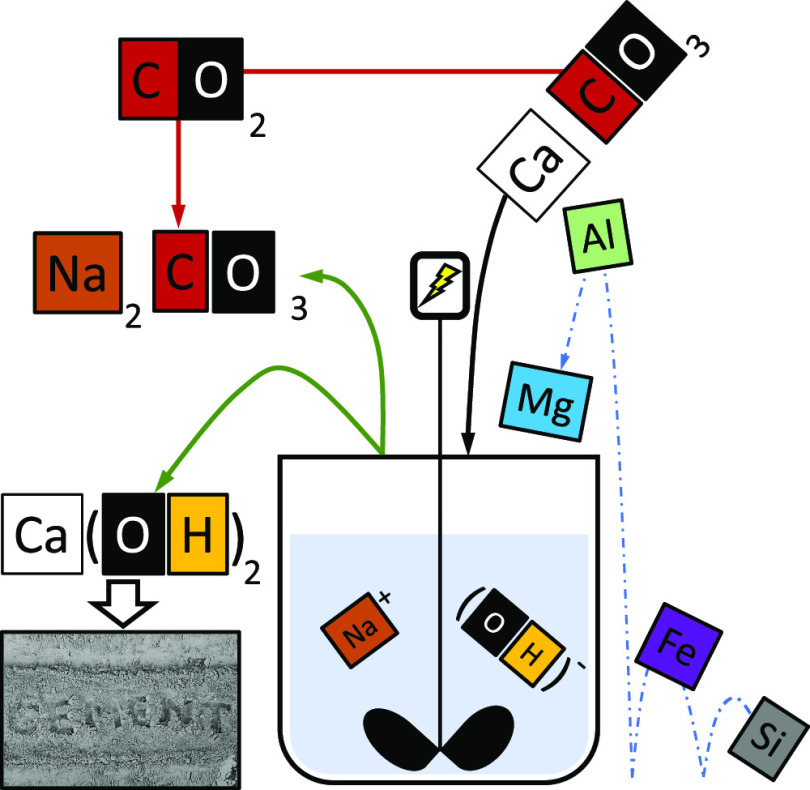

Decarbonizing calcium carbonate (CaCO_3_) is a crucial step for a wide range of major industrial processes and materials, including Portland cement (PC) production. Apart from the carbon footprint linked to fuel combustion, the process CO_2_ embodied within CaCO_3_ represents the main concern for the sustainability of production. Our recent works demonstrated that it is possible to avoid both the fuel and process CO_2_ by reacting CaCO_3_ with aqueous NaOH and obtain Ca(OH)_2_ and Na_2_CO_3_·*x*H_2_O (*x* = 0 and 1). This present study provides a further understanding of the process by testing different raw calcareous sources. A high decarbonization (∼80%) of CaCO_3_ was achieved for silica-rich chalk, whereas a lower extent was obtained (∼50%) for limestone. To understand the difference in their reaction behavior, the effect of impurities was studied. The effects of the major impurities (Si, Al, and Fe) were found to be marginal, which is advantageous to process industrial grade materials, while the morphology of the raw materials presents a significant impact. The applicability of our decarbonization technology was also demonstrated on magnesite (MgCO_3_).

## Abstract 2

The impurities within the raw materials did not affect the efficiency of the CaCO_3_ chemical decarbonization for sustainable cement production.

## 1 Introduction

The calcination of calcium carbonate to obtain lime (CaCO_3_ → CaO + CO_2_) is currently considered one of the major contributors to the global CO_2_ emissions due to both the large global demand^1^ and the specific carbon footprint (1.0–1.8 kg_CO2_/kg_CaO_).^2^ The Portland cement (PC) industry is currently utilizing the largest portion of calcined limestone, with a global market size of 4 Gt PC per year,^3^ which makes the cement industry responsible for about 8% of the total CO_2_ emissions worldwide^4^ and 12–15% of global industry energy use.^5^

The calcination of calcium carbonate usually involves two distinct emission sources: process- and fuel-derived CO_2_. The former arises from the calcination stoichiometry (0.44 kg_CO2_/kg_CaCO3_),^6^ while the latter is linked to the combustion of the hydrocarbon fuels to attain the required pyro-processing temperatures (∼900 °C^2,7^ for lime production and 1500 °C^5^ for PC production). Although the fuels represent the largest portion of the overall economic operating costs for both lime and cement industries,^8^ the process CO_2_ represents the biggest challenge for their sustainable production. The process CO_2_ accounts for the majority of the CO_2_ emissions from the limestone calcination step, and several solutions have been proposed: Carbon Capture & Storage (CCS) technologies,^9^ the switch to sustainable fuels,^7,10^ and the development of low-calcium cements.^11^ Currently, the CCS technologies are believed to have the highest potential to decarbonize the cement industry. They might be classified as pre- or post-combustion; while the former options require a deep modification of the current design, the latter ones are usually retrofittable with the conventional lime and cement plants.^100−13^ Among the CCS technologies available, the CO_2_ removal through reaction with monoethanolamine (MEA) solutions appears to be the most developed solution so far,^9^ despite the high operational costs linked to the regeneration of the solvent.^14^ The use of waste as fuel is a common practice already, accounting for a significant replacement of fossil sources in the EU depending on the country;^15^ the selection and pretreatment of the waste are essential to ensure a good quality of the manufactured product. The use of alternative binders with a lower Ca content would reflect a production with limited process CO_2_ emissions. In addition, the reuse of several types of waste as substitutes to the conventional cement chemistry might play a determining role in the waste disposal challenge.^16^ These potential solutions are all based on the high-temperature calcination process.

In contrast, we recently proposed an alternative technology that exploits the chemical interaction between CaCO_3_ and NaOH in an aqueous system under ambient conditions^17^ so that the high-temperature calcination process itself can be avoided. On the other hand, the application of such a decarbonization route would require a drastic modification of the current plants, with high investment costs associated. Moreover, the significant usage of NaOH would raise concerns in terms of embodied CO_2_ and Cl_2_ emissions from the chlor-alkali process.^18^ Despite this, since the chlor-alkali process is mainly sustained by electric energy,^18^ the NaOH production would be expected to be carbon-neutral by 2050 if the goals set up during the Paris agreement in 2015 will be met.^19^ Regarding Cl_2_, its disposal might partially be performed through recycling into Cl-based cements, such as alinite.^20,21^ As shown in eq 1, the alternative decarbonization route leads to the synthesis of Ca(OH)_2_, which can also be converted to CaO in mild conditions through dehydroxylation, Ca(OH)_2_ → CaO + H_2_O,^7^ while sequestrating the process CO_2_ into a stable mineral form, i.e., Na_2_CO_3_·*x*H_2_O (*x* = 0 and 1).
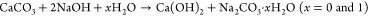
1

For cement clinker production, high temperatures are still required for the formation of clinker phases,^1^ but such conditions can be achieved through electrical heating^5^ and E-fuels,^22^ concentrated solar power,^23^ or combustion of waste/biomass.^24^

Our previous work^17^ demonstrated the feasibility of the proposed decarbonization technique on reagent grade calcium carbonate and a particular calcium carbonate (chalk) source. For the industrial applications, variabilities in the raw material source are inevitable, which would affect the process.^1,25^ In the present study, two different industrial grade materials with significantly different compositions, a limestone and a chalk (same as in our previous work^17^), are considered.

Limestone and chalk are mainly calcium carbonate but are generally different both at macro- and microscopic levels,^26^ and this allows us to assess the efficiency of our process for varying calcareous sources. Due to the nature of our aqueous process, it is envisaged that the different average porosities (chalk, >25%;^27^ limestone, <10%^28^) will play a crucial role in the present investigation. A higher or lower permeability and diffusion of foreign elements (e.g., Na^+^) could make the difference between more and less reactive materials for the scope of this technology; generally, the higher the porosity (ϕ), the higher the permeability (*k*).^29^

In this work, deeper insight into the mechanisms ruling the reaction is discussed by considering different calcareous sources with varying contents of impurities. The different efficiencies registered could suggest an important effect of varying contents of impurities (Si, Al, Fe, and Mg) in the reactants. For this reason, the effect of each of them was isolated in reagent grade binary systems with increasing CaCO_3_ content, and the outcomes are thoroughly discussed and justified by cross-linking analyses. Finally, to detect those parameters, i.e., impurity content or particle morphology, which could mostly influence the reaction efficiency, reagent grade systems simulating the compositions of the chalk and limestone were tested. The authors are well aware that additional considerations must be done in terms of process design (including the hazards linked to the high concentration of the NaOH solutions used), energy consumption, and carbon balance before even considering a process scale-up; despite this, the outcomes reported here would still provide a valid baseline for further considerations to be done.

## 2 Experimental Section

### 2.1 Materials

The present work used the following commercial grade chemicals: Sigma-Aldrich CaCO_3_ (≥99%), Honeywell Fluka NaOH (≥97%), Sigma-Aldrich purum p.a. white quartz as a SiO_2_ source (≥95%), Acros Organics extra pure Al_2_O_3_ (99%), Fisher Chemical pure Fe_2_O_3_ (99.85%), and Honeywell MgCO_3_ basic (MgO > 40%). Their solubilities in water and methanol are reported in Table S1. The industrial grade limestone and chalk used in the present work were provided by CEMEX; their oxide compositions (Table 1) were obtained via X-ray fluorescence (XRF).

**Table 1:** Oxide Composition (wt %) of the Limestone and Chalk Used, Together with the Respective Loss on Ignition (LOI) Values Gained by XRF

	CaCO_3_	SiO_2_	Al_2_O_3_	Fe_2_O_3_	MgCO_3_	others (K, Ti, P)	LOI (%)
limestone	94.4	1.2	0.3	0.5	3.2	<1.0	42.3
chalk	74.2	19.9	2.8	1.0	0.7	<1.5	29.4

Considering the loss on ignition values of 29.4% for the chalk and 42.3% for the limestone, the Ca and Mg content is reported as CaCO_3_ and MgCO_3_ rather than CaO and MgO, respectively. The limestone presented a higher CaCO_3_ content (94.4 wt %) with respect to the chalk (74.2 wt %). A significant silica content (19.9 wt %) was detected within the chalk, whereas more MgCO_3_ (3.2 wt %) were found in the limestone. Traces of Fe_2_O_3_ were also detected in both materials. To compare the results between the reagent and industrial grade calcareous sources, the raw materials were manually sieved below 38 μm. As reported in Figure S1, the PSD analysis revealed average diameters (Dx50) of 6.0 and 8.5 μm for the chalk and limestone, respectively; most of the reagent grade CaCO_3_ solid particles were in the range of 20–40 μm, with a negligible amount of smaller (5 μm) ones.

### 2.2 Characterization Techniques

#### 2.2.1 X-ray Diffraction (XRD)

X-ray diffraction (XRD) was used to identify the reaction products. The measurements were performed using a Bruker D2 PHASER desktop X-ray diffractometer in Bragg–Brentano geometry, with a Cu-Kα radiation source running at 30 kV and 10 mA, a one-dimensional LYNXEYE detector, and a 1 mm divergence slit. Powdered samples were loaded onto 2.5 cm-diameter and 1 mm-deep sample holders. Each pattern was recorded between 5° and 80° 2θ, with a step size of 0.02° at 0.5 s per step, with the stage rotating at 15 rpm. Qualitative phase identification was carried out using the Highscore-Plus software and PDF-42019 database.

#### 2.2.2 Thermogravimetry (TG/DTG)

Thermogravimetric analysis (TG) was carried out on the reaction products. Approximately 40 mg of sample was analyzed on a PerkinElmer TG 4000 from 30 to 800 °C at a heating rate of 10 °C/min with a 40 mL/min N_2_ flow. The sample was then held at 800 °C for 1 h to ensure complete loss of CO_2_ from CaCO_3_ while maintaining Na_2_CO_3_ without melting or decomposing. To identify evolving gases, a Hiden mass spectrometer (HPR-20 GIC EGA) was used to record the signals for H_2_O and CO_2_. As shown in eq 2, the extent of reaction α was calculated from the weight losses in the temperature ranges corresponding to the thermal decomposition of Ca(OH)_2_ (310–470 °C^30^) and CaCO_3_ (560–800 °C^31^). The terms w%_[phase]_ and MW_phase_ refer to the weight loss registered in TG and the molecular weight of the substance considered, respectively. The content of Na_2_CO_3_·H_2_O could similarly be estimated from mass loss in the temperature range of 50–130 °C.^32^ The possible measurement error was estimated by analyzing the same sample six times under the same condition as ±0.16 wt % for Na_2_CO_3_·H_2_O, ±0.10 wt % for Ca(OH)_2_, and ±0.16 wt % for CaCO_3_.
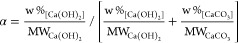
2

#### 2.2.3 Scanning Electron Microscopy (SEM)

Scanning electron microscopy with energy-dispersive X-ray spectroscopy (SEM–EDX; Hitachi TM3030) was used for the microstructural analysis of the starting powders and reaction products at a 15 kV voltage and working distance of approximately 9 ± 0.2 mm. This was fitted with the Bruker Quantax Energy Dispersive X-ray Spectrometer for compositional analysis through BSE detectors. The reaction products were mounted, in powder form, in epoxy resin without crushing and left to harden for 72 h. The analysis surface was ground manually with progressively finer abrasives, up to a 1 μm finish,^33^ and further polished by using diamond pastes of 6, 3, 2, 1, and 0.25 μm (MetPrep). The samples then underwent a three-step carbon coating and were back-loaded to a metallic holder. Electrically conductive silver paint (RS Components) was applied at the interface between the metallic base epoxy resin to ensure the sufficient conductivity and, therefore, good quality of the SEM micrographs.

#### 2.2.4 X-ray Fluorescence (XRF)

X-ray fluorescence was used to quantify the elemental composition of the unreacted and reacted solids; the measurement was performed through a PW4404 AMG Analytical spectrometer, with an Ar/CH_4_ gas flow and a Rh X-ray tube. Samples were crushed and milled to obtain a particle size within the range of 100–250 μm. The milled materials were dried at 110 °C until a constant weight was achieved. The powder was mixed in the fusion vessel with a flux, lithium tetraborate (Li_2_B_4_O_7_), at a 1:10 sample-to-flux weight ratio and then fired at 1270 ± 15 °C for 12 min upon swirling. The detection limit of the XRF analysis depends on both the sample preparation and the atomic number *Z* of the targeted elements. Generally, detection limits of 20–1000, 5–10, and 1–20 μg·g^–1^ are reported for low-, medium-, and high-*Z* elements, respectively.^34^ For this reason, the detection of Mg may be affected by the instrumental error since it belongs to the second group in the periodic table (*Z* = 12).

### 2.3 Reaction Procedure

Despite the specific conditions stated below for each targeted investigation, all the experiments were conducted according to the same experimental procedure. Upon dissolution of NaOH in water at known molalities (m), the solutions were left to cool down to room temperature. The solids were dried at 35 °C overnight prior reaction to remove the weakly bound water, which might slightly affect the overall NaOH concentration used. The reaction was carried out in a 250 mL PTFE beaker to avoid corrosion that may be caused by the hyper-alkaline NaOH solutions, and a stirring rate of 1050 rpm was ensured through a Heidolph R2020 overhead mixer equipped with a PTFE centrifugal stirrer shaft (40 mm diameter). The reaction was carried out under ambient/laboratory conditions (*T* ≈ 20 °C) for a residence time of 300 s. To remove the unreacted NaOH after reaction, all the samples discussed above were washed with methanol for further 300 s. Considering the solubility of NaOH in methanol at 20 °C (238 g/L^35^) and the amount of NaOH in the starting mixtures, the complete removal of NaOH was ensured by choosing a methanol-to-NaOH weight (g/g) ratio of 4. Given the negligible solubility of the targeted phases Ca(OH)_2_,^36^ Na_2_CO_3_·H_2_O,^37^ Na_2_CO_3_,^37^ CaCO_3_,^38^ SiO_2_,^39^ Al_2_O_3_,^40^ Fe_2_O_3_,^40^ and MgCO_3_^40^ in organic solvents, no variation of the solid mixture should have occurred upon washing with methanol. Finally, the reaction products obtained from all the experiments discussed in the present work were collected on a Whatman Grade 1 (90 mm) filter paper using vacuum-assisted Büchner funnel filtration, dried in an oven at 35 °C for 2 h, weighed, ground, and sieved below 63 μm for characterization.

#### 2.3.1 Decarbonization of Industrial Grade Calcareous Materials

The industrial grade chalk and limestone were reacted with NaOH at the NaOH/CaCO_3_ molar ratio of 3 based on the CaCO_3_ contents obtained by XRF of the materials (Table 1). This ratio was previously found to positively influence the reaction yield.^17^ The systems were tested at increasing water-to-solid weight ratio, which in return decreases the NaOH molalities in the aqueous solution (4 m–40 m). The detailed starting mix compositions are reported in Table 2.

**Table 2:** Compositions Inspected for the Limestone (L Series) and Chalk (C Series) and the Corresponding NaOH/CaCO_3_ (mol/mol) and H_2_O/Solids (w/w) Ratios

sample ID	H_2_O (wt %)	NaOH (wt %)	feed material (wt %)	NaOH/CaCO_3_ (mol/mol)	H_2_O/feed material (w/w)	NaOH (mol· kg_H2O_^–1^)
L_w/s_0.7	24.8	40.3	35.0	3.0	0.7	40.6
L_w/s_1.0	31.8	36.4	31.7	3.0	1.0	28.6
L_w/s_1.5	41.2	31.4	27.4	3.0	1.5	19.0
L_w/s_2.0	48.2	27.7	24.1	3.0	2.0	14.3
L_w/s_3.0	58.2	22.4	19.4	3.0	3.0	9.6
L_w/s_5.0	69.9	16.1	14.0	3.0	5.0	5.7
C_w/s_0.6	24.4	35.1	40.5	3.0	0.6	36.0
C_w/s_0.7	27.4	34.0	38.7	3.0	0.7	31.1
C_w/s_1.0	34.8	30.4	34.8	3.0	1.0	21.8
C_w/s_1.5	44.5	25.8	29.6	3.0	1.5	14.5
C_w/s_2.0	51.7	22.5	25.8	3.0	2.0	10.9
C_w/s_3.0	61.6	18.0	20.4	3.0	3.0	7.3
C_w/s_5.0	72.7	12.7	14.5	3.0	5.0	4.4

#### 2.3.2 Effect of Impurities (Si, Al, Fe, and Mg)

The effect of common impurities in the chalk and limestone (i.e., Si, Al, Fe, and Mg) on the decarbonization reaction was studied. Binary systems of CaCO_3_-SiO_2_, CaCO_3_-Al_2_O_3_, CaCO_3_-Fe_2_O_3_, and CaCO_3_-MgCO_3_ were tested using reagent grade chemicals at varying proportions (Table 3) to simulate the oxide compositions of the chalk and limestone (Table 1). This would isolate the effect of each main impurity and allow for the assessment of their effects on the overall reaction.

**Table 3:** Composition of Starting Solid Mixtures (wt %) of the Binary Systems and NaOH/CaCO_3_ (mol/mol) and H_2_O/Solids (w/w) Ratios Used in the Reactions

sample ID	νCaCO_3_ (wt %)	νSiO_2_ (wt %)	νAl_2_O_3_ (wt %)	νFe_2_O_3_ (wt %)	νMgCO_3_ (wt %)	NaOH/CaCO_3_ (mol/mol)	H_2_O/solids (w/w)
reference	100.0	0.0				3.9	4.0
SiO_2__1.0%	99.0	1.0				4.0	4.0
SiO_2__2.9%	97.1	2.9				4.0	3.9
SiO_2__4.8%	95.2	4.8				4.0	3.8
SiO_2__6.5%	93.5	6.5				4.0	3.7
SiO_2__9.1%	90.9	9.1				4.1	3.6
SiO_2__13.1%	86.9	13.1				4.0	3.5
SiO_2__20.0%	80.0	20.0				4.0	3.2
Al_2_O_3__1.0%	99.0		1.0			4.0	4.0
Al_2_O_3__2.0%	98.0		2.0			4.0	3.9
Al_2_O_3__2.9%	97.1		2.9			4.0	3.9
Al_2_O_3__4.8%	95.2		4.8			4.0	3.8
Fe_2_O_3__0.5%	99.5			0.5		4.0	4.0
Fe_2_O_3__1.0%	99.0			1.0		4.0	4.0
Fe_2_O_3__1.5%	98.5			1.5		4.0	3.9
Fe_2_O_3__2.0%	98.0			2.0		4.0	3.9
Fe_2_O_3__4.8%	95.2			4.8		4.0	3.8
Fe_2_O_3__9.1%	90.9			9.1		4.0	3.6
MgCO_3__0.6%	99.4				0.6	4.0	4.0
MgCO_3__1.0%	99.0				1.0	4.0	3.9
MgCO_3__1.5%	98.5				1.5	4.0	4.0
MgCO_3__4.8%	95.2				4.8	4.0	3.8
MgCO_3__9.1%	90.9				9.1	4.0	3.6
MgCO_3__16.7%	83.3				16.7	4.0	3.3

Prior to the reaction, the minerals were ground, sieved below 38 μm, and dried at 35 °C overnight to ensure the homogeneous particle size and limited presence of water, which might lower the overall NaOH concentration used. The 10 m NaOH solutions were prepared to ensure a NaOH/CaCO_3_ molar ratio of 4 for all the samples. Despite the decreasing w/s ratios used at higher additions of SiO_2_, Al_2_O_3_, Fe_2_O_3_, and MgCO_3_ (Table 3), previous investigations revealed that such a parameter would not affect the reaction efficiency in the ranges considered here. A high content of water was chosen for the starting mixture to avoid the agglomeration of solids and, therefore, error.

To study the effect of the coexisting impurities, reagent grade chemicals were also blended according to the proportions reported in Table 4, simulating the industrial grade chalk and limestone used in the present work (Table 1).

**Table 4:** CaCO_3_, SiO_2_, Al_2_O_3_, and MgCO_3_ Contents for the Chalk_R.G. and Limestone_R.G. Powders Simulating the Industrial Grade Chalk and Limestone

sample ID	νCaCO_3_ (wt %)	νSiO_2_ (wt %)	νAl_2_O_3_ (wt %)	νFe_2_O_3_ (wt %)	νMgCO_3_ (wt %)
Chalk_R.G.	75.2	20.2	2.8	1.1	0.7
Limestone_R.G.	94.9	1.2	0.3	0.4	3.2

These mixtures simulating chalk and limestone were tested in the same way as in the testing of the industrial grade materials (Section 2.3.1), including the starting mix composition (Table 5).

**Table 5:** Summary of the Conditions Used for the Reaction of the Reagent Grade Powders Simulating the Industrial Grade Materials Tested and Discussed in Section 2.3.1

sample ID	H_2_O (wt %)	NaOH (wt %)	solids (wt %)	NaOH/CaCO_3_ (mol/mol)	H_2_O/solids (w/w)	NaOH (mol/L)
L_R.G._w/s_0.7	24.8	40.3	34.9	3.0	0.7	40.6
L_R.G._w/s_1.0	31.9	36.5	31.7	3.0	1.0	28.6
L_R.G._w/s_1.5	41.2	31.4	27.4	3.0	1.5	19.0
L_R.G._w/s_2.0	48.2	27.7	24.1	3.0	2.0	14.4
L_R.G._w/s_3.0	58.2	22.4	19.4	3.0	3.0	9.6
L_R.G._w/s_5.0	69.9	16.1	14.0	3.0	5.0	5.7
C_R.G._w/s_0.6	24.4	35.2	40.4	3.0	0.6	36.0
C_R.G._w/s_0.7	27.3	34.0	38.7	3.0	0.7	31.1
C_R.G._w/s_1.0	34.8	30.4	34.8	3.0	1.0	21.8
C_R.G._w/s_1.5	44.5	25.8	29.7	3.0	1.5	14.5
C_R.G._w/s_2.0	51.7	22.5	25.8	3.0	2.0	10.9
C_R.G._w/s_3.0	61.6	18.0	20.5	3.0	3.0	7.3
C_R.G._w/s_5.0	72.7	12.7	14.5	3.0	5.0	4.4

## 3 Results and Discussion

### 3.1 Industrial Grade Calcareous Materials

**Figure 1:**
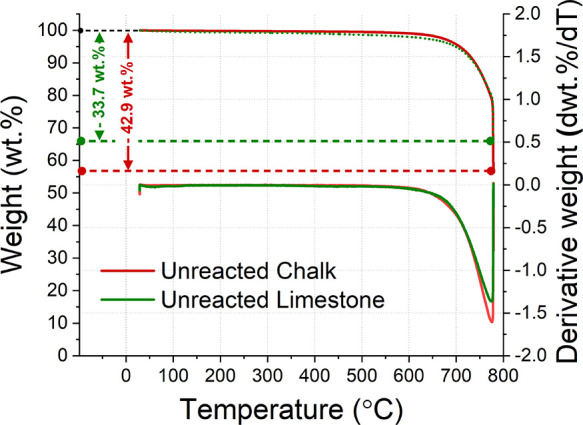
TG/DTG analysis performed for both the limestone and chalk studied.

The TG analysis (Figure 1) performed on the unreacted raw calcareous materials confirmed the XRF quantification of CaCO_3_ reported in Table 1, with slight variations: CaCO_3_ contents of 73.7 wt % (33.7% of weight loss) and 96.8 wt % (42.9% of weight loss) for the chalk and limestone, respectively.

**Figure 2:**
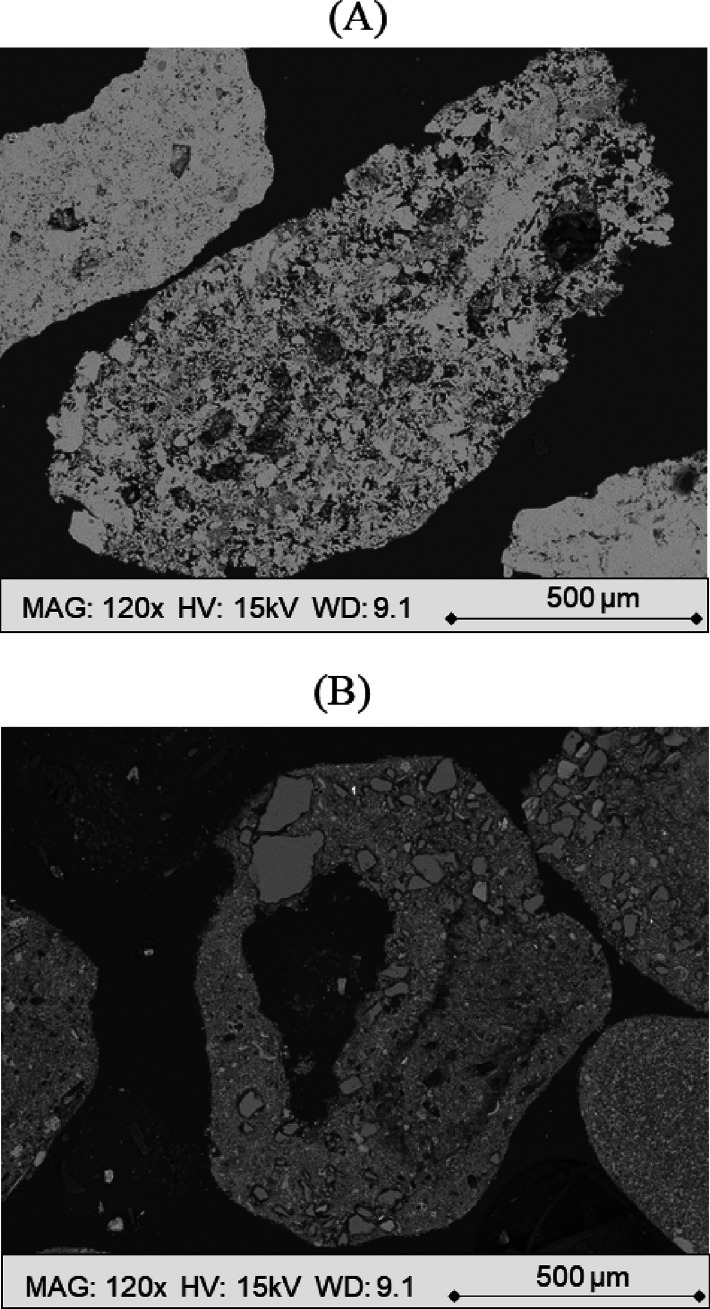
SEM micrographs of large-sized chalk (A) and limestone (B) unreacted particles at a magnification and working distance of 120× and 9.1 mm, respectively.

The SEM analysis was used to assess the overall differences of the two materials tested. First, large particles of unreacted chalk and limestone were selected through manual sieving, mounted in epoxy resin, and analyzed (Figure 2A,B), revealing their morphological characteristics. At first sight, the chalk appeared more porous than the limestone, with a more irregular surface.

**Figure 3:**
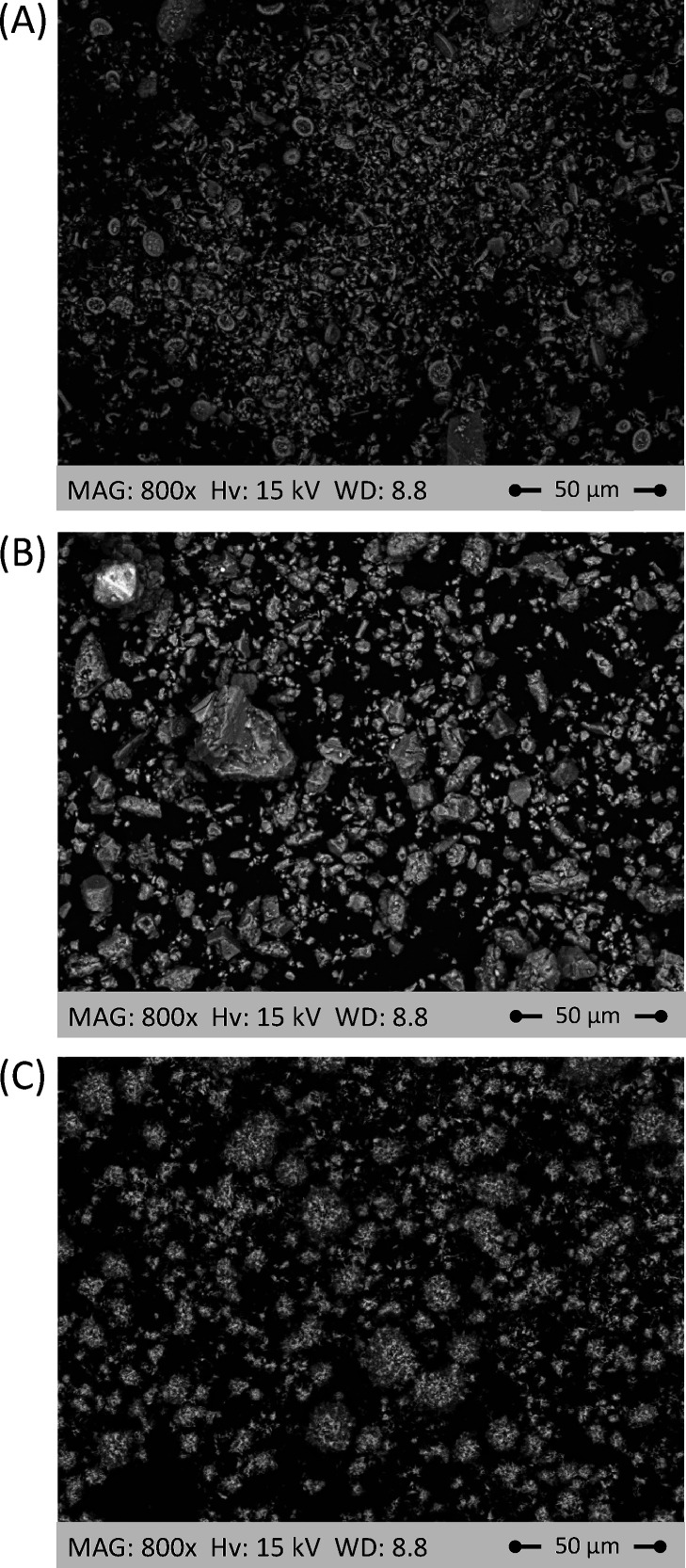
SEM micrographs of chalk (A), limestone (B), and reagent grade CaCO_3_ (C) unreacted particles sieved below 38 μm at a magnification and working distance of 800× and 8.8, respectively.

The SEM analysis was repeated on the smaller particles (below 38 μm), not epoxy-mounted, but attached to a conductive carbon adhesive tape. The conventional coccolith-like^41^ shaped CaCO_3_ crystals are observed for the chalk, visible as light gray circles in Figure 3A. In contrast, a heterogeneous morphology could be observed for the limestone, whose CaCO_3_ crystals were showing larger beads of scalenohedral^42^ and smaller beads of cubic^43^ geometry (Figure 3B). For completion, the SEM analysis was performed on the reagent grade CaCO_3_ used for comparison with industrial grade materials; as shown in Figure 3C, it was mainly composed of thin plates arranged in spherical agglomerations.

### 3.2 Decarbonization of Industrial Grade Calcareous Materials

**Figure 4:**
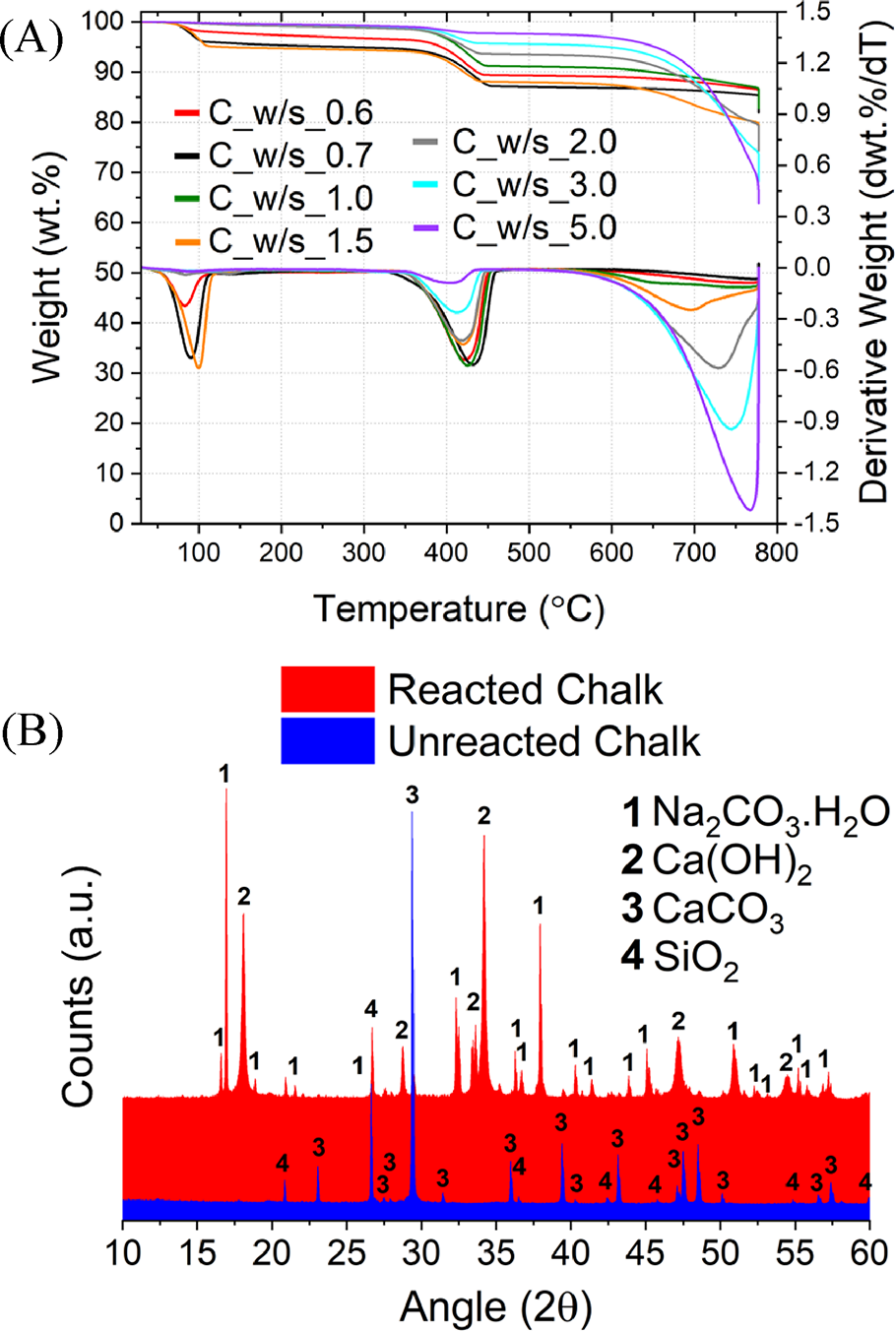
TG and DTG trends observed for all the chalk samples (A), together with the XRD patterns for C_w/s_0.7 and unreacted chalk powders (B).

The industrial grade limestone and chalk were reacted with NaOH solutions at a constant NaOH/CaCO_3_ molar ratio of 3 with increasing water-to-feed material ratio, as reported in Table 2. As shown in the TG/DTG data in Figure 4A, the dehydroxylation of Na_2_CO_3_·H_2_O and Ca(OH)_2_ between 50 and 130 °C and between 310 and 470 °C, respectively, and the decarbonization of the remaining CaCO_3_ between 560 and 800 °C could be detected for the chalk samples.

The comparison between the XRD patterns for the unreacted and reacted chalk reported in Figure 4B confirmed the outcomes from the TG analysis. Indeed, only Na_2_CO_3_·H_2_O, Ca(OH)_2_, and CaCO_3_ could be identified as reaction products. Given the relatively low content in impurities, their eventual dissolution could not be assessed through XRD analysis despite the fact that the decreasing intensity of the main peak linked to SiO_2_ (26.6° 2θ) would suggest that the dissolution of silica would occur upon the reaction. To assess that, XRF analysis was conducted on the sample C_w/s_0.7, showing the highest capability to convert CaCO_3_ to products. Given that the solids would undergo a weight increase upon decarbonization reaction,^44^ the ratio between the weight percentage of Ca and the specific foreign element was taken as the mass balance for the system. These values are reported in Table 6, which show the mass balance performed prior to and upon the reaction. Apparently, all the foreign elements were dissolving at a certain extent upon the reaction, as outlined by the lower Si/Ca, Al/Ca, Fe/Ca, and Mg/Ca ratios with respect to the initial values. Silica was the main component in the chalk, after CaCO_3_, and the SEM micrograph reported in Figure S2A shows an irregular geometry that might be the cause of the dissolution observed.

**Table 6:** Mass Balance for Each Element Prior to and upon the Reaction, Expressed as Si/Ca, Al/Ca, Fe/Ca, and Mg/Ca Ratios

	initial				final			
	Si/Ca	Al/Ca	Fe/Ca	Mg/Ca	Si/Ca	Al/Ca	Fe/Ca	Mg/Ca
C_w/s_0.7	3.1 × 10^–01^	5.0 × 10^–02^	2.4 × 10^–02^	6.9 × 10^–03^	1.4 × 10^–01^	3.6 × 10^–02^	1.8 × 10^–02^	5.4 × 10^–03^
L/w/s_2.0	1.5 × 10^–02^	4.2 × 10^–03^	7.5 × 10^–03^	2.5 × 10^–02^	3.2 × 10^–02^	9.9 × 10^–03^	1.3 × 10^–02^	3.4 × 10^–02^

**Figure 5:**
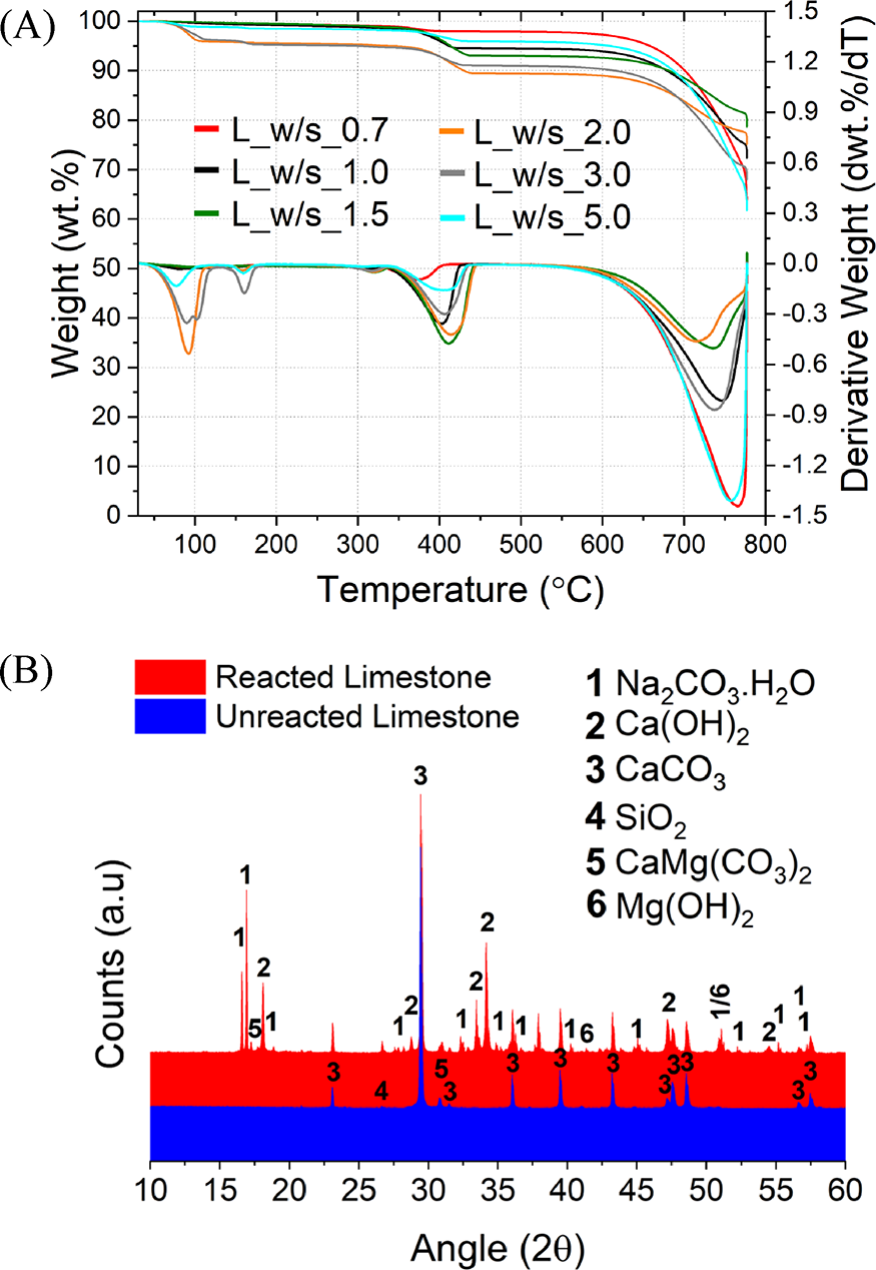
TG and DTG trends observed for all the limestone samples (A), together with the XRD patterns for L_w/s_3.0 and unreacted limestone powders (B).

The TG analysis performed on the limestone (Figure 5A) revealed additional signals in the ranges of 140–200 and 250–350 °C for the samples reacted at higher w/s ratios; also, the sample L_w/s_3.0 showed an anomalous double peak in the region of 50–130 °C.

Given the higher MgCO_3_ content within the limestone (Table 1), the signals between 250 and 350 °C were likely attributed to the dehydration of brucite Mg(OH)_2_.^45^ The signals between 140 and 200 °C may be linked to the dehydration of monohydrocalcite CaCO_3_·H_2_O,^46^ suggesting its formation at generally higher water proportions. The double peak in the temperature region of 50–130 °C, with maxima at 85 and 102 °C, might potentially be due to the two-step dehydration of Na_2_CO_3_·H_2_O,^47^ which could not be observed for any other samples discussed here. The comparison between the XRD patterns of unreacted limestone and the sample L_w/s_3.0 (Figure 5B) supported the TG analysis. Slight traces of dolomite CaMg(CO_3_)_2_ could be observed for the limestone both prior to and upon the reaction, in accordance with the higher Mg content within the powders identified by XRF (Table 1). A similar intensity of the peaks of CaCO_3_ suggests the limited reaction of CaCO_3_ to form Ca(OH)_2_, and weak intensities of brucite could also be observed. Again, the eventual dissolution of the foreign elements could be investigated by exploiting the XRF analysis performed on the sample showing the highest conversion extent (L_w/s_2.0). In contrast with the products from the reacted chalk, the Si/Ca, Al/Ca, Mg/Ca, and Fe/Ca ratios were decreasing for the sample L_w/s_2.0 upon the reaction (Table 6). Such an unexpected outcome would suggest that a portion of the calcium initially introduced would dissolve upon the reaction. Likely, the dissolution of dolomite to form brucite (Figure 5B) would contribute to the decreasing final Ca content within the solids. Potentially, given the relatively low content in Mg (Table 1), and therefore, dolomite, some of the calcite in the limestone would dissolve too. However, such a fact did not allow us to assess the eventual dissolution of the foreign elements Si, Al, Mg, and Fe for the limestone.

**Figure 6:**
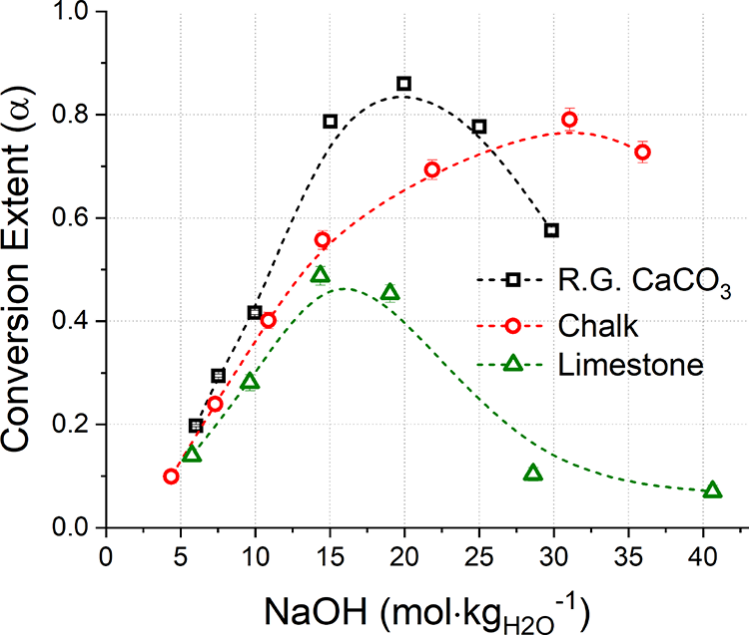
Overall efficiency of the systems at increasing NaOH molalities for the reacted chalk, limestone, and reagent grade CaCO_3_, calculated by substituting the TG data into eq 2.

Based on the amount of Ca(OH)_2_ and CaCO_3_ estimated through the TG data, the extent of the decarbonization reaction was assessed using eq 2. The outcomes of the assessment are plotted against the concentration of NaOH in Figure 6.

Since all samples have a constant NaOH/CaCO_3_ ratio, the concentration of NaOH also represents the amount of H_2_O in the system: the higher the NaOH concentration, the less the amount of H_2_O. Despite the higher purity, the chalk appears to be much more reactive than the limestone, resulting in the higher extent of decarbonization (Figure 6). All materials, including the reagent grade CaCO_3_, were showing a bell-shaped trend: the chalk indicated the maximum extent of decarbonization of 0.79 ± 0.02 at a NaOH concentration of 31.1 m, while the limestone achieved the maximum extent of decarbonization of 0.49 ± 0.02 at a NaOH concentration of 14.3 m. The reagent grade CaCO_3_ showed the same trend observed for the other materials, registering the maximum extent of decarbonization of 0.86 ± 0.03 at a 20 m NaOH concentration.

### 3.3 Effect of Impurities: Individual Effect

**Figure 7:**
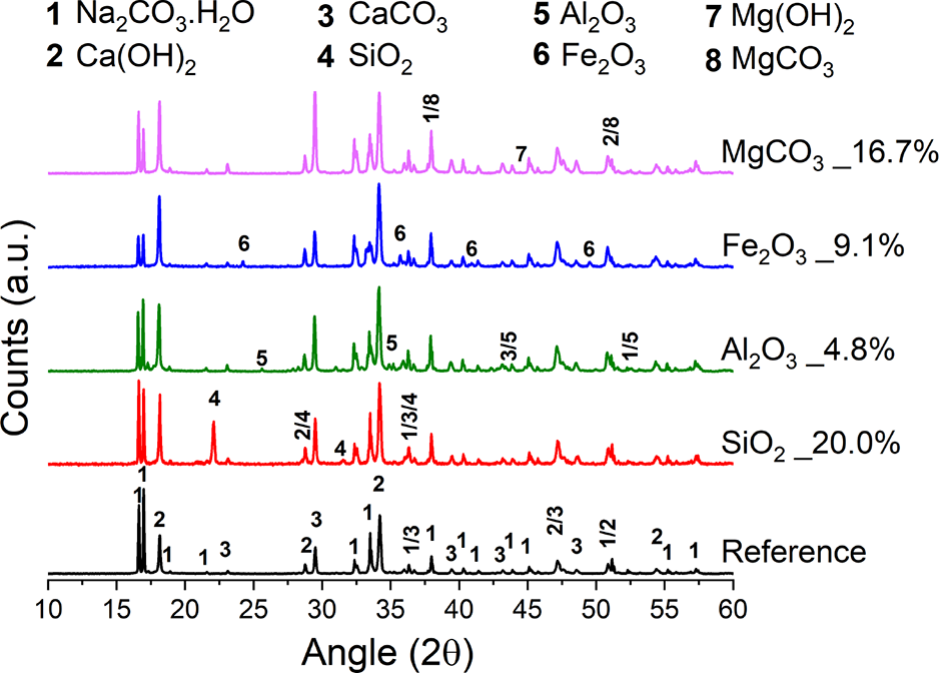
XRD patterns of the reference, SiO_2__20.0%, Al_2_O_3__4.8%, Fe_2_O_3__9.1%, and MgCO_3__16.7% samples with main crystalline phases highlighted.

The effects of major impurities were studied on the decarbonization reaction of the industrial grade materials. To isolate the effect of any individual impurity, reagent grade materials were used to test binary systems of CaCO_3_-SiO_2_, CaCO_3_-Al_2_O_3_, CaCO_3_-Fe_2_O_3_, and CaCO_3_-MgCO_3_, with varying proportions of impurities (Table 3). It is worth highlighting that the oxides of the targeted element were used here, rather than the corresponding minerals, such as corundum, hematite, and dolomite. For all systems, solid reaction products were recovered and underwent TG and XRD analysis. The quantification of the foreign elements was performed through XRF for those specimens showing the highest and lowest decarbonization efficiencies in each series. Representative XRD data for the solid reaction products of each system are shown in Figure 7. The XRD analysis confirms the occurrence of the decarbonization reaction with clear reflection peaks of Ca(OH)_2_ and Na_2_CO_3_·H_2_O.

**Figure 8:**
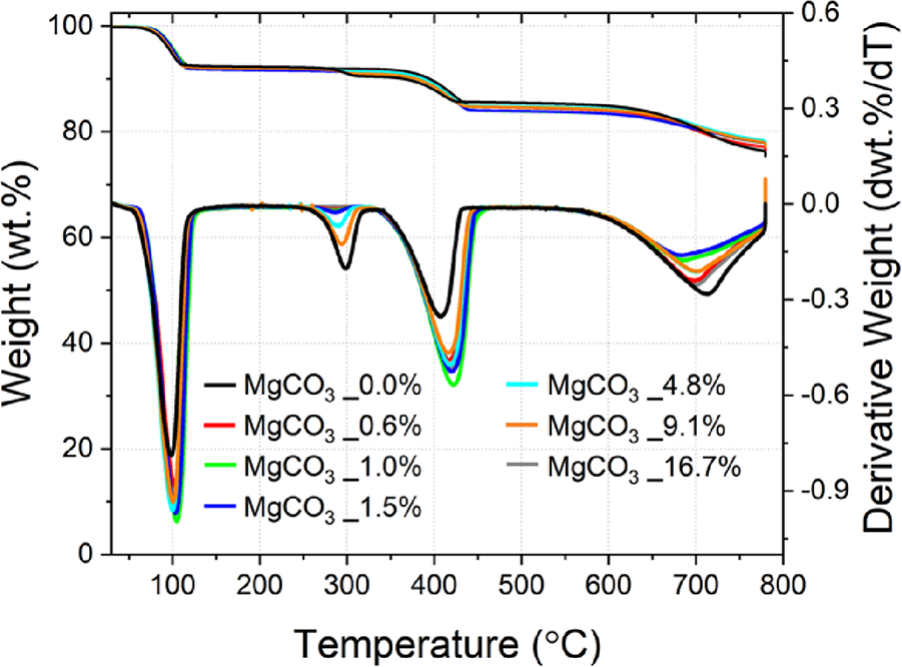
TG and DTG trends observed for the binary system CaCO_3_:MgCO_3_ reported in Table 3.

The TG analysis confirmed the presence of the main phases Na_2_CO_3_·H_2_O, Ca(OH)_2_, and CaCO_3_, with no additional signals detected for the SiO_2__*n*%, Al_2_O_3__*n*%, and Fe_2_O_3__*n*% series, as reported in Figure S3. Together with the XRD data just discussed (Figure 7), the absence of secondary reactions for these systems was confirmed except brucite formation in the CaO-MgCO_3_ system. Differently, the weight losses observed between 250 and 350 °C for the MgCO_3__*n*% series (Figure 8) could likely be referring to the dehydration of brucite, as also confirmed by the XRD pattern of the sample MgCO_3__16.7% in Figure 7. Accordingly, the intensity of the signal was increasing at higher MgCO_3_ proportions initially blended with CaCO_3_.

The quantification of Na_2_CO_3_·H_2_O, Ca(OH)_2_, and CaCO_3_, based on the TG data, allowed us to estimate the compositions of Na and Ca in the solid reaction product using eqs 3 and 4, respectively.
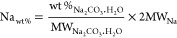
3
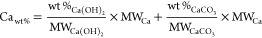
4

**Figure 9:**
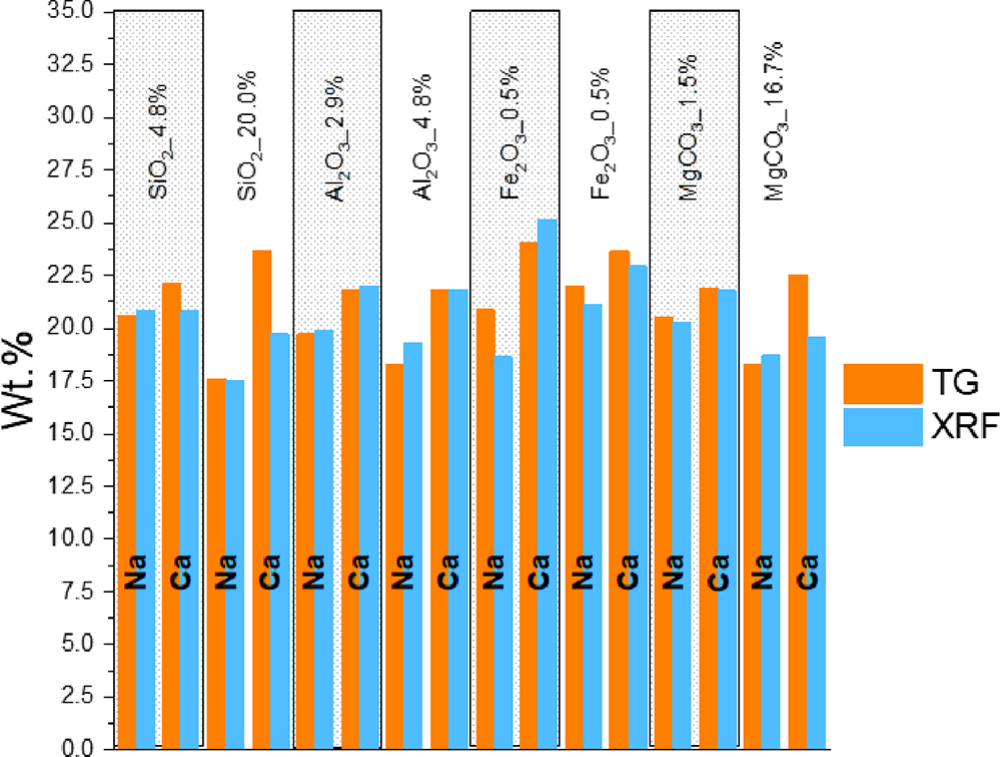
Effect of impurity contents on the amount of Na and Ca in the solid reaction products (data obtained by XRF and TG): the type and quantity of the impurity are indicated at the top.

The contents of Na and Ca gained from the TG analysis and respective calculations are in good agreement with the XRF data recorded on targeted samples, as reported in Figure 9. A slight overestimation of the Ca-containing species (CaCO_3_ and Ca(OH)_2_) was gained from the TG analysis of the samples SiO_2__20% and MgCO_3__16.7%, with respect to XRF (Figure 9). Despite that, the theoretical Ca content calculated by considering the Na_wt %_ in the sample, referring to the product Na_2_CO_3_·H_2_O, was higher than the value gained from XRF. Potentially, the higher Si and Mg content in the samples SiO_2__20% and MgCO_3__16.7%, respectively, could be the cause of a slight underestimation of Ca through XRF. However, despite the fact that these values were slightly off, the overall good correspondence between the TG and XRF data was likely suggesting a high reliability of the phase quantification, and linked conversion efficiency α, performed through TG analysis.

**Figure 10:**
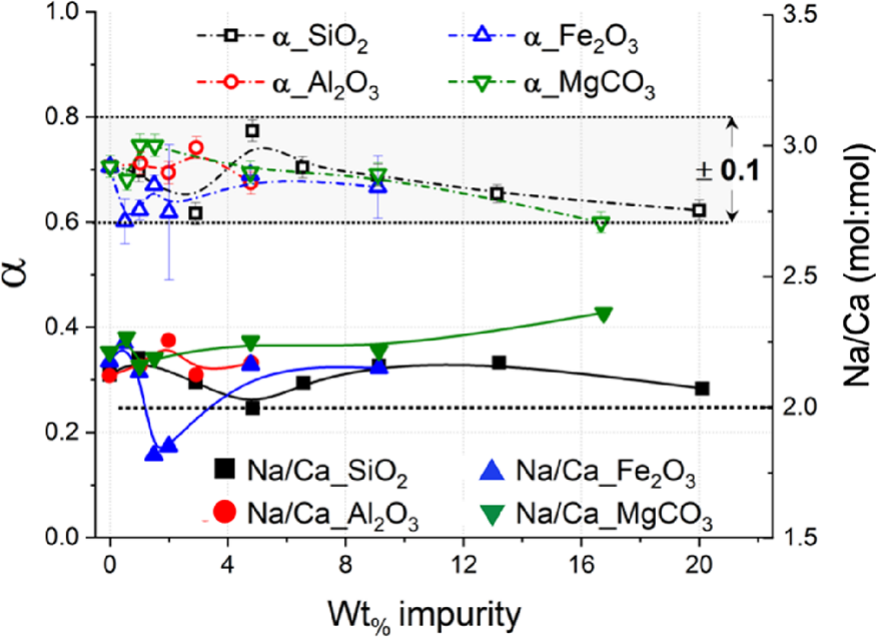
Conversion extent (α) and Na/Ca (mol %/mol %) ratios, calculated through eqs 5 and 6, for all the binary systems shown here. The lines only work as a guide for the eye.

Based on the TG data, the amounts of Na_2_CO_3_·H_2_O, Ca(OH)_2_, and CaCO_3_ were calculated for each solid reaction product to estimate the extent of decarbonization reaction as shown in Figure 10.

Moreover, to gain a better understanding of the precipitation of the main products Ca(OH)_2_ and Na_2_CO_3_·H_2_O or Na_2_CO_3_, Na_mol %_ and Ca_mol %_ were first expressed in eqs 5 and 6, respectively.
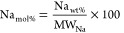
5
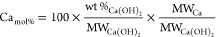
6While Na_mol %_ is linearly linked to eq 3 by the molecular weight of Na (MW_Na_), the Ca_mol %_ here only refers to the fraction of Ca present in the system as Ca(OH)_2_. By considering these values, it was possible to highlight a Na/Ca molar ratio slightly above 2 for all the samples discussed (Figure 10), apart from the specimens Fe_2_O_3__1.5% and Fe_2_O_3__2.0%. Since the stoichiometric ratio of the reaction products Ca(OH)_2_ and Na_2_CO_3_·H_2_O or Na_2_CO_3_ is 1 (eq 1), the Na/Ca molar ratio should be 2. The slightly exceeding values might indicate higher kinetics for the precipitation of Na_2_CO_3_·H_2_O or Na_2_CO_3_ with respect to Ca(OH)_2_. Another possibility might relate to a partial loss of Ca(OH)_2_ upon dissolution, leading to higher Na/Ca molar ratios, but this is unlikely since the solubility of Ca(OH)_2_ is about 220 and 205 times lower than those of Na_2_CO_3_·H_2_O and Na_2_CO_3_, respectively (Table S1).

The efficiency of the reaction varies with the type and amount of the impurity but generally remains at around α = 0.71 with possibly a slight decrease when the amount of impurity increases over 10 wt %. Specifically, increasing contents of Al_2_O_3_ and MgCO_3_ were not significantly affecting the reaction efficiency, which remained constant throughout the ranges investigated.

The reactions of the systems with SiO_2_ and Fe_2_O_3_ were prepared twice to experimentally confirm the nonlinear trends detected, and thus, their data in Figure 10 indicate the standard deviation. The efficiency of the reaction appears to slightly increase at 4.8 wt % SiO_2_ content. Such a silica content may increase the efficiency of the decarbonization, but additional investigation is required to confirm and elucidate the trend. With Fe_2_O_3_ impurity, the decarbonization reaction appeared to be reduced at 0.5 and 2.0 wt %, but that might be due to the significant experimental error (Figure 10).

**Figure 11:**
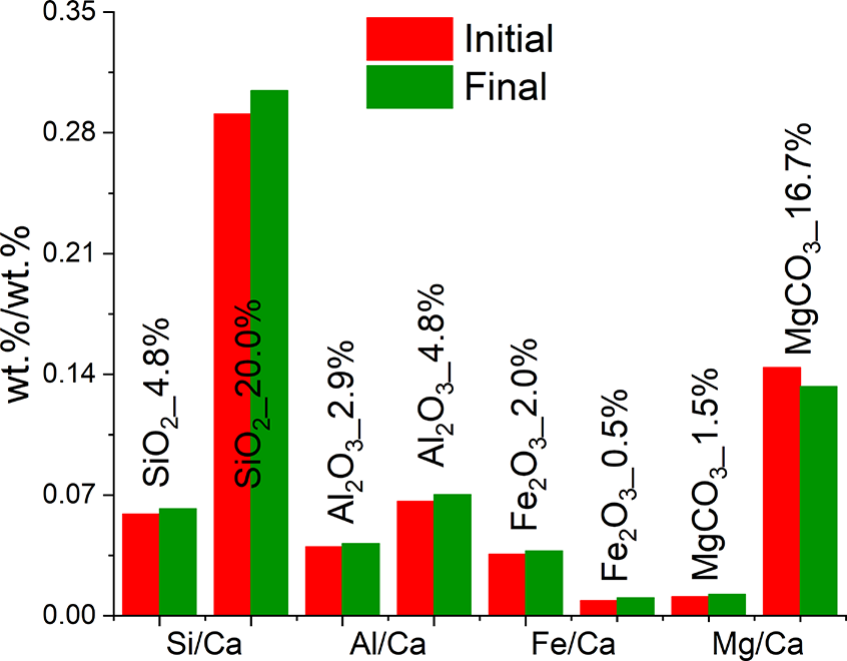
Change in Si/Ca, Al/Ca, Fe/Ca, and Mg/Ca weight ratios between the initial solid mixtures and the solid reaction products.

Based on the initial composition and the XRF data of the reaction products, Si_wt %_/Ca_wt %_, Al_wt %_/Ca_wt %_, Fe_wt %_/Ca_wt %_, and Mg_wt %_/Ca_wt %_ ratios were calculated for the selected systems, as shown in Figure 11. The mass balances prior to and upon the reaction are not indicating substantial variations for the elements considered, suggesting that the dissolution of those foreign species was not significantly occurring. Such an observation was contradicting the results obtained from the industrial grade chalk and limestone, since silica appeared to dissolve at some extent in those systems (Table 6). Apparently, the reagent grade silica was less reactive than the one within the industrial grade materials, and the reason might be linked to the more irregular surface of the latter (Figure S2). The slight overestimation of Mg and Fe for the samples MgCO_3__1.5% and Fe_2_O_3__0.5%, respectively (Figure 11), could possibly be linked to the low Mg and Fe contents and, therefore, the instrumental error.

The obtained results show that the individual effects of the main impurities of the chalk and limestone tested in the present work are likely minimal on the decarbonization reaction. The relatively short residence time did not allow for the dissolution of SiO_2_, Al_2_O_3_, and Fe_2_O_3_ in the experiments conducted, except the reaction of MgCO_3_ to form Mg(OH)_2_. These phases remain with the solid reaction products upon washing with methanol (Table S1). In terms of application, for instance, for the cement production, the presence of SiO_2_, Al_2_O_3_, Fe_2_O_3_, and brucite would not represent a problem, as these are the same “impurities” in raw materials used for traditional cement production. Indeed, the presence of silicates and aluminates is crucial for the synthesis of clinker phases.^1^

### 3.4 Effect of Impurities: Combined Effects

**Figure 12:**
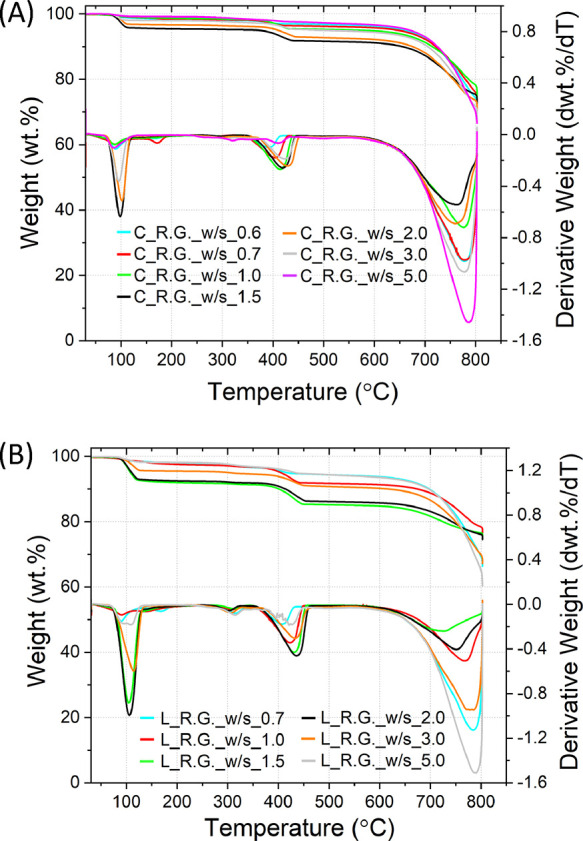
TG and DTG trends observed for the C_R.G._w/s_n (A) and L_R.G._w/s_n (B) samples.

To investigate the combined effect of the impurities, samples were prepared by blending reagent grade materials to simulate the oxide compositions detected for the chalk and limestone (Table 1). Only the main impurity constituents (>1 wt %) detected in the industrial grade materials, such as SiO_2_, Al_2_O_3_, Fe_2_O_3_, and MgCO_3_, were considered and blended with reagent grade CaCO_3_. The compositions of the reagent grade systems considered are reported in Table 4. These mixtures were tested at increasing H_2_O/solids ratios, corresponding to decreasing NaOH molarity, as shown in Table 5; the TG/DTG analysis performed on the reaction products is provided in Figure 12A,B.

Similar to the previous experimental results, the recovered solid reaction products indicated weight loss events attributed to the presence of Na_2_CO_3_·H_2_O (50–130 °C), Ca(OH)_2_ (310–470 °C), and CaCO_3_ (560–800 °C). Both systems indicated a minor formation of monohydrocalcite, reflected by weak weight losses in the region of 150–200 °C for the samples reacted at a water-to-solid ratio of 0.7 (Figure 12A,B). A small weight loss observed in the region of 250–350 °C is likely referred to the dehydration of brucite; more intense signals were detected for L_R.G. with respect to C_R.G., in line with the higher initial MgCO_3_ content (Table 4).

**Figure 13:**
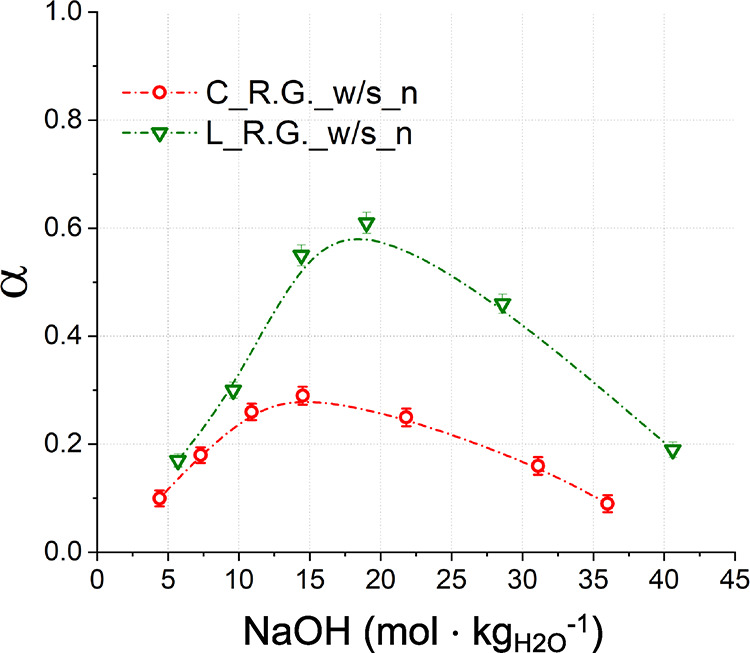
Overview of the conversion extent α registered for the C_R.G. and L_R.G. samples at increasing NaOH molarities.

Based on the amount of Ca(OH)_2_ and CaCO_3_ estimated from the TG data, the extent of decarbonization reaction is assessed and indicated in Figure 13.

As reported, the decarbonization for the C_R.G. solids was significantly lower than that for L_R.G. for all the NaOH molarities tested. Although the effects of individual impurities are not clearly identified in the former section, it appears that the efficiency of decarbonization reaction becomes less when the amount of the impurity is larger, as the C_R.G. system has a larger proportion of the total impurity. This suggests either the potential synergetic effect of the impurities or the effect of the NaOH solution used (as NaOH/CaCO_3_ was set to be 3.0 for all reactions, the C_R.G. system used less NaOH than the same weight of the L_R.G. system). These results are also in contrast with those obtained from the industrial grade calcareous materials (Figure 6), suggesting that the morphology of the materials has a significant impact on the decarbonization reaction in the condition investigated in the present work.

**Figure 14:**
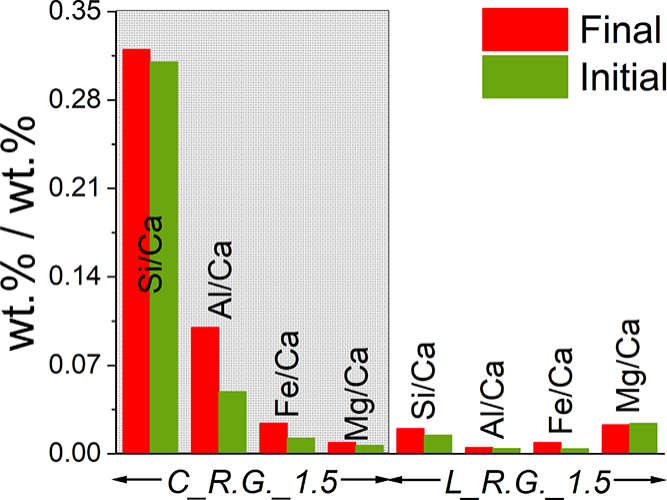
Mass balances for Ca, Si, Al, and Mg expressed as wt % Si/Ca, Al/Ca, Fe/Ca, and Mg/Ca ratios, respectively.

XRF analysis was also conducted on the samples showing the highest efficiency of decarbonization, i.e., C_R.G._w/s_1.5 (20.0 M NaOH) and L_R.G._w/s_1.5 (15.0 M NaOH). As reported in Figure 14, the proportion of the impurity components in the reaction products remains constant upon the reaction when assuming that the dissolution of CaCO_3_ and Ca(OH)_2_ is negligible in the alkaline solutions at the residence times considered here.^48^ In other words, the XRF data suggest that the impurities are not likely dissolving in the alkaline solution upon the reaction.

### 3.5 Discussion

In the decarbonization reaction proposed, the formation of Ca(OH)_2_ and Na_2_CO_3_·H_2_O/Na_2_CO_3_ should theoretically be 1:1 in moles (eq 1); that would theoretically correspond to a Na/Ca molar ratio of 2, with Ca only referring to Ca(OH)_2_. However, slightly more enhanced precipitation of Na_2_CO_3_·*x*H_2_O was observed with respect to Ca(OH)_2_ when the effect of MgCO_3_ was investigated (Figure 10). That might be explained by considering the fact that the surface of calcite is statistically 27% denser in positively charged (Ca^2+^) sites than negatively charged (CO_3_^2–^) ones,^49^ resulting in an enhanced tendency to interact with cationic species, such as Na^+^. Accordingly, a higher affinity should be present for Na^+^ than OH^–^ in the system investigated, which may have resulted in the slightly more enhanced precipitation of Na_2_CO_3_·*x*H_2_O than Ca(OH)_2_. The introduction of MgCO_3_ would likely provide additional negative binding sites (CO_3_^2–^) interacting with Na^+^, leading to a more enhanced precipitation of Na_2_CO_3_·*x*H_2_O than Ca(OH)_2_.

Decarbonization of industrial grade calcareous materials indicated the higher decarbonization efficiency in the chalk compared with the limestone. This could be likely explained by the higher surface area registered for the chalk, providing a larger number of CO_3_^2–^ and Ca^2+^ binding sites for the interaction with Na^+^ and OH^–^ ionic species in the liquid bulk.

Additionally, the larger content of silica within the chalk (Table 1) might potentially play a significant role in terms of reactivity, since it would provide additional Si^4+^ and O^2–^ sites that could interact with the ions in the liquid bulk. However, a lower efficiency in decarbonizing CaCO_3_ was generally registered for the reagent grade mixture simulating the chalk (Table 4), as reported in Figure 13, suggesting that higher contents of impurities would hinder the reaction at parity of the calcareous source used.

**Figure 15:**
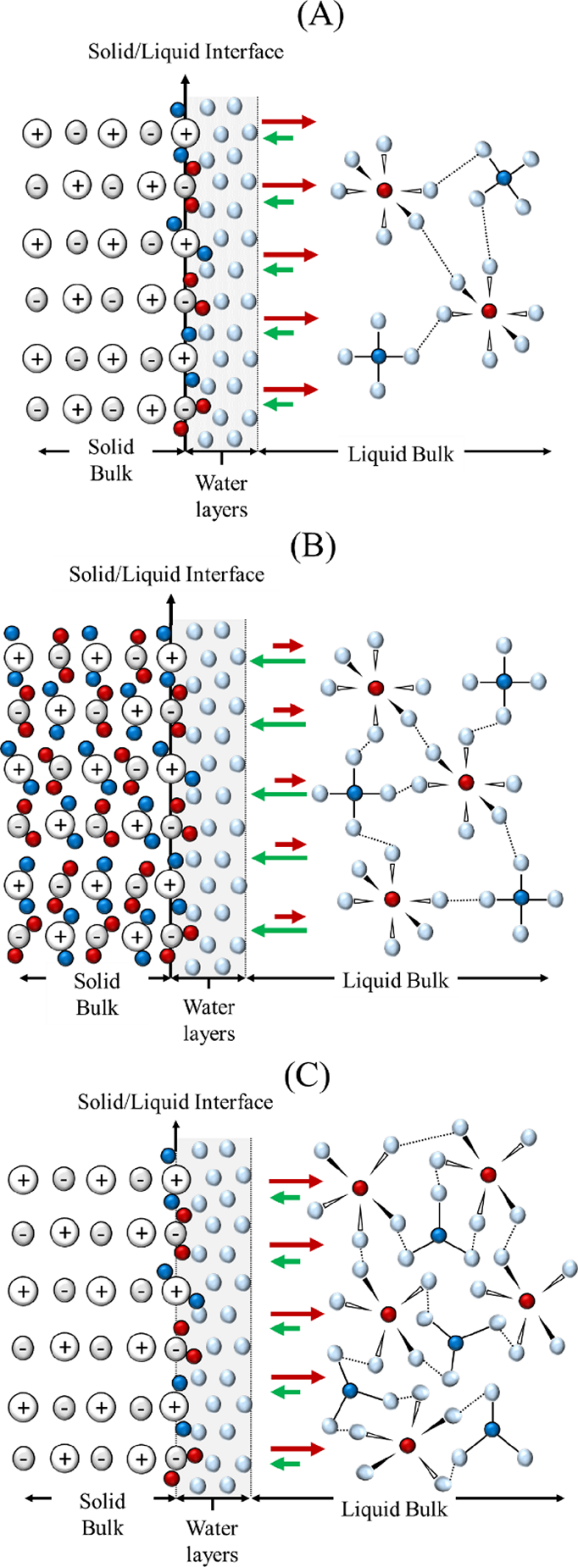
Schematic representation of the interactions occurring within the liquid and solid bulk considered for the study, where the red, dark blue, and light blue colors refer to Na^+^, OH^–^, and H_2_O, respectively. Each Na^+^ and OH^–^ ion is surrounded by a number of water molecules, forming the solvation shell, depending on the NaOH concentration.^52^ The hydrogen bonds between the Na^+^ and OH^–^ ions and the water molecules forming the solvation shell and between water molecules of adjacent ions are also displayed. The solid/liquid interface is highlighted in light gray, outlining the attachment of those four layers of water, whereas the green and red arrows qualitatively represent the attractive and repulsive forces between the ions in solution and the solid surface. Cases A, B, and C refer to low, medium/optimal, and high NaOH concentrations, respectively.

The bell-shaped profile was obtained in the decarbonization efficiency with different NaOH concentrations, both with industrial grade and reagent grade calcareous materials (Figures 6 and 13). To understand the reduced decarbonization efficiency observed at relatively low and high NaOH molarities, it is useful to consider the situation at the solid–liquid interface. In solution, the surface binding sites of the solids are readily saturated with strongly adsorbed layers of water^50^ up to four layers below the surface, as shown in Figure 15A. Moreover, it is well known that a diffuse double layer would form at the interface of the solid and liquid bulk upon incorporation within the solid of a charged species.^51^ The double layer is electrically charged positively and negatively when adsorbed CO_3_^2–^ and Na^+^ are considered, respectively, to ensure the electroneutrality of the surface. Apart from the steric encumbrance linked to the layers of water attached to the surface of calcite, this layer would also contribute to the overall energetic barrier to overcome for the uptake of Na^+^ and CO_3_^2–^ to occur. With a low NaOH concentration, the chemical potential in the liquid bulk would not be sufficient to overcome the energetic barrier for the nucleation and precipitation to occur. However, the limited reaction efficiencies suggest that the low NaOH concentration was enough to at least saturate the surface binding sites of the solid calcite beneath the layer of water as indicated in Figure 15A. When the NaOH concentration increases, as shown in Figure 15B, its chemical potential in the liquid bulk becomes sufficient both for the saturation of the surface binding sites and for promoting the nucleation and precipitation, diffusing toward the inner part of the solids. However, when the NaOH concentrations become too high, as shown in Figure 15C, the nucleation and precipitation of the reaction product become harder, likely given the too high activity of the ions. Moreover, the higher viscosity of the NaOH solution might have lowered the contact with the solid reactants.

Figure 15

## 4 Conclusions

An alternative no-combustion CaCO_3_ decarbonization route, involving the production of Ca(OH)_2_ and direct capture of the process CO_2_ into Na_2_CO_3_·*x*H_2_O, on industrial grade calcareous materials was investigated. The reaction efficiency was higher for a type of chalk rich in SiO_2_ (19.9 wt %) compared with limestone mostly composed of CaCO_3_ (94.4 wt %). The maximum decarbonization efficiency α of 0.79 was achieved for the chalk reacting with 31.1 M NaOH, while a value of 0.49 was obtained for the limestone with 14.3 M NaOH. The higher irregularity of the chalk surface, likely leading to a larger number of readily available binding sites, is believed to be the main reason behind this efficiency difference. In fact, additional experiments performed with reagent grade reactants highlighted that the solid solution simulating the limestone (rich in CaCO_3_) was more reactive if the same calcareous source was considered.

The bell-shaped trend observed in the decarbonization efficiency with increasing NaOH molarities was also discussed. Likely, low NaOH concentrations would allow only for the saturation of the surface binding sites of calcite, while the subsequent nucleation and precipitation would be increased at higher concentrations (a higher chemical potential) of NaOH within the liquid. However, the lower conversion efficiency observed at too high NaOH molarities was likely linked to the enhanced viscosity of the liquid bulk, hindering the ionic mobility and further interaction with the solid reactants.

The effect of the major impurities was assessed individually with reagent grade materials, and only slight fluctuations in the reaction efficiency were observed at increasing contents of SiO_2_, Al_2_O_3_, Fe_2_O_3_, and MgCO_3_. The elemental analysis of the powders prior to and upon the reaction suggested negligible dissolution of SiO_2_, Al_2_O_3_, and Fe_2_O_3_, while MgCO_3_ reacts to form brucite Mg(OH)_2_.

In conclusion, the present study demonstrated the feasibility of the chemical CaCO_3_ decarbonization route on different industrial sources without combustion. The effect of microscopic morphology and surface of the CaCO_3_ source was more significant than that of impurities. This unconventional route for the decarbonization of limestone could minimize the CO_2_ emissions both from the conventional calcination of CaCO_3_ and combustion of fuels, simultaneously sequestrating CO_2_ in a stable carbonate mineral form. It has a great potential, with further understanding and development, toward a sustainable future of relevant industries.

## Supporting Information Available

Table S1: solubility (g·L^–1^) of the relevant solid phases in water and methanol at 25 °C and 1 atm; Figure S1: particle size distribution (PSD) performed for both the limestone and chalk studied; Figure S2: SEM micrographs of the silica crystals within the chalk and limestone, together with the reagent grade silica; Figure S3: TG/DTG trends observed for the binary systems CaCO_3_:SiO_2_, Al_2_O_3_:SiO_2_, and Fe_2_O_3_:SiO_2_, reported in Table 4 (PDF)
