# Supplementary material for: Effect of Impurities on the Decarbonization of Calcium Carbonate Using Aqueous Sodium Hydroxide
Source: ACS Sustain Chem Eng. 2022 Aug 26;10(36):11913–25. doi: 10.1021/acssuschemeng.2c02913 (PMC9472277; doi:10.1021/acssuschemeng.2c02913)
Supplement: Supplementary file 1 — sc2c02913_si_001.pdf [file sc2c02913_si_001.pdf]

Supporting Information

"Effect of impurities on the decarbonisation of calcium carbonate using aqueous sodium hydroxide"

Marco Simoni, Theodore Hanein*, Chun Long Woo, John Provis and Hajime Kinoshita*.*

University of Sheffield, Department of Materials Science & Engineering, S1 3JD, Sheffield, United Kingdom
* Corresponding author: t.hanein@sheffield.ac.uk, h.kinoshita@sheffield.ac.uk, marco.simoni.w@gmail.com

Number of Pages: 3

Number of Tables: 1

Number of Figures: 3

Chemical	*Solubility in water at 25 ⁰C, 1 atm (g L^{-1})*	*Solubility in methanol at 25 ⁰C, 1 atm (g L^{-1})*
SiO_2	*n.s.*[54]	*n.s.*[54]
Al_2O_3	*n.s.* [40]	*n.s.* [40]
Fe_2O_3	*n.s.* [40]	*n.s.* [40]
$MgCO_3$	*$1.1\cdot10^{-3}$* [55]	*n.s.* [55]
$CaCO_3$	*$1.4\cdot10^{-4}$* [38]	*n.s.* [38]
$Ca(OH)_2$	*1.5* [36]	*n.s.* [36]
Na_2CO_3	*307.0* [37]	*n.s.* [37]
$Na_2CO_3.H_2O$	*330.0* [37]	*n.s.* [37]

Table S1. Solubility (g L^{-1}) of the relevant solid phases in water and methanol at 25^{0}C and 1 atm; n.s. stands for not soluble.

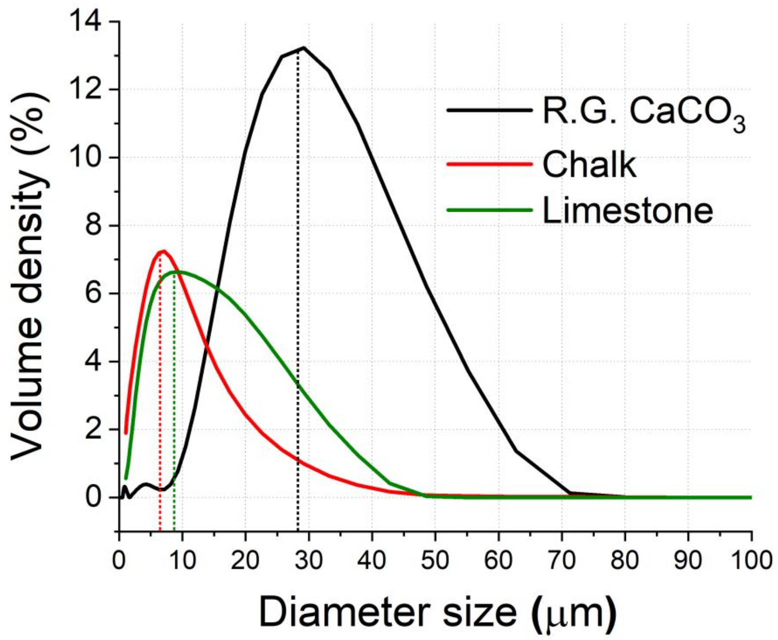

Figure S1. Particle Size Distribution (PSD) performed for both the limestone and the chalk studied.

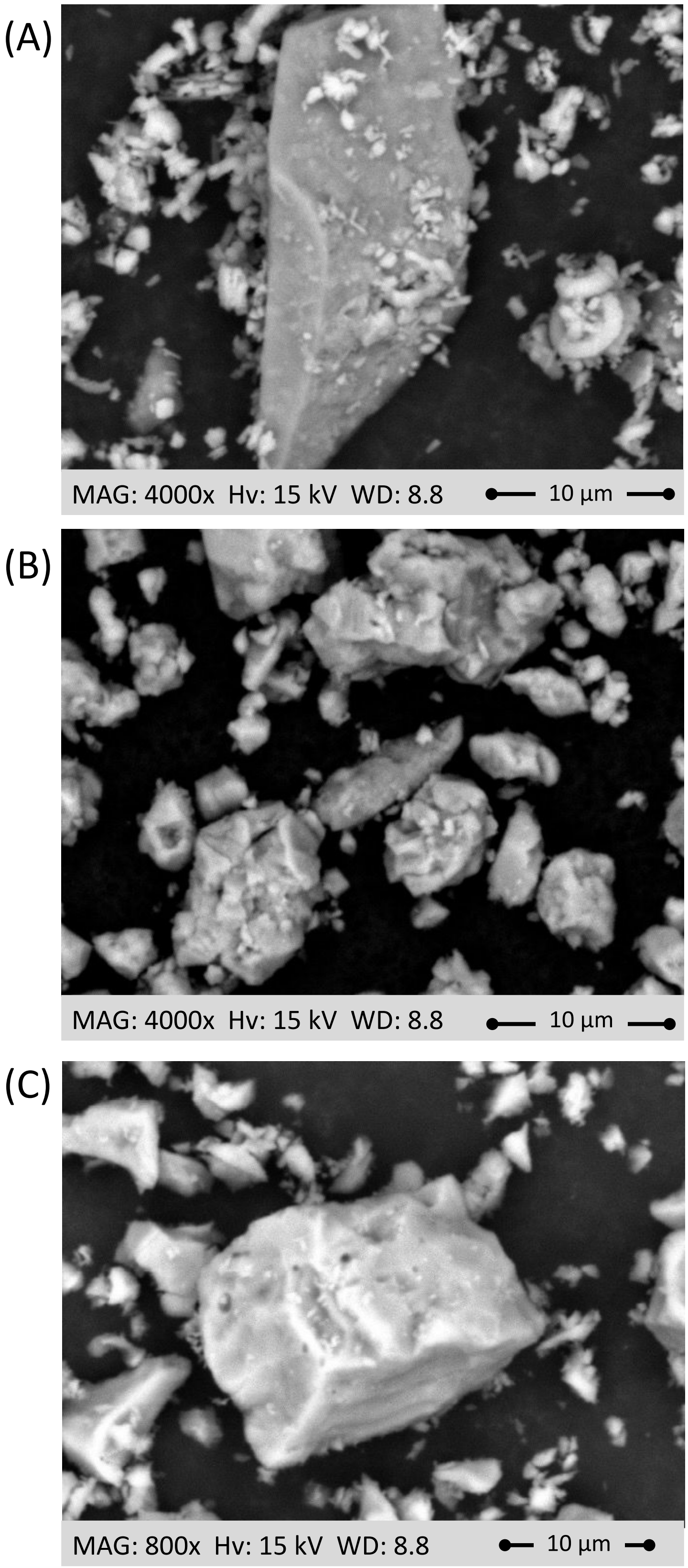

Figure S2. SEM micrographs of the silica crystals within the chalk (A) and limestone (B), together with the reagent grade silica (C); the analyses were conducted at 15 kV, magnification and working distance of 800 and 8.8, respectively.

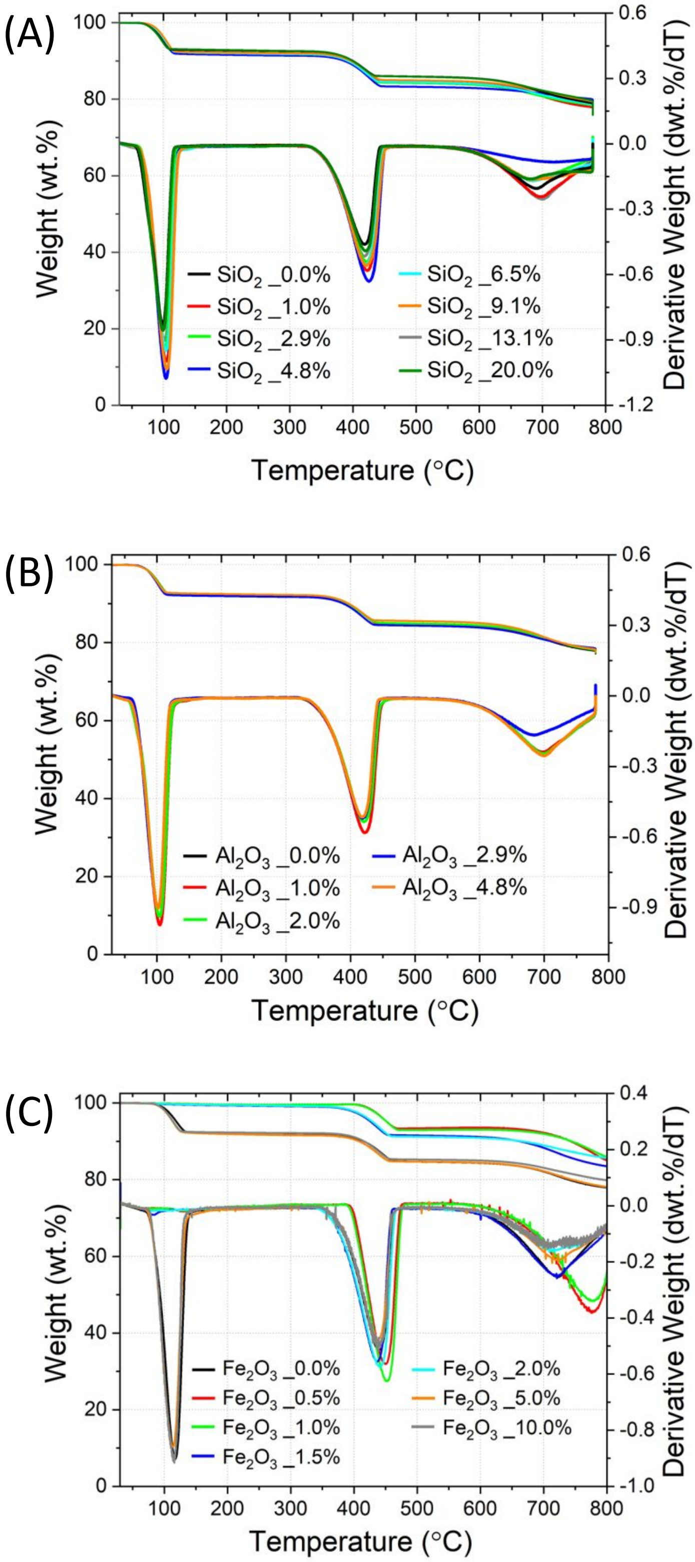

Figure S3. TG/DTG trends observed for the binary system $CaCO_3$:SiO_2 (A), Al_2O_3:SiO_2 (B), and Fe_2O_3:SiO_2 (C), reported in Table 4.
